# Supplementary material for: Impaired cortico-striatal excitatory transmission triggers epilepsy
Source: Nat Commun. 2019 Apr 23;10:1917. doi: 10.1038/s41467-019-09954-9 (PMC6478892; doi:10.1038/s41467-019-09954-9)
Supplement: Supplementary file 1 — Supplementary Information [file 41467_2019_9954_MOESM1_ESM.pdf]

Supplementary information

Impaired Cortical Excitatory Transmission Triggers Epilepsy

Hiroyuki Miyamoto, Tetsuya Tatsukawa, Atsushi Shimohata, Tetsushi Yamagata, Toshimitsu Suzuki, Kenji Amano, Emi Mazaki, Matthieu Raveau, Ikuo Ogiwara, Atsuko Oba-Asaka, Takao K. Hensch, Shigeyoshi Itohara, Kenji Sakimura, Kenta Kobayashi, Kazuto Kobayashi, Kazuhiro Yamakawa.

Supplementary Figures: 15
Supplementary Videos: 4

Supplementary Fig. 1. **Characterization of SWDs in *Stxbp1*$^{+/-}$ mice.**
a, Spontaneous myoclonic seizures (3 out of 43 mice, left) and generalized tonic-clonic seizures (2 of 43 mice, right) were observed at a low rate in *Stxbp1*$^{+/-}$ mice. Convulsive seizures while awake were occasionally observed [once per several hours during 30 hours of observation (10 hours × 3 days)]. **b,** Simultaneous representative SSC-ECoG (top) and CPu-LFP (bottom) recordings. SWD was defined as more than three spike-and-waves with negative (SSC-ECoG) or positive (CPu- LFP) spikes. **c,** An example of SSC-ECoG positive polyspike discharges not counted as SWDs (top). Most discharges had a slower frequency (~3 Hz) than SWDs. There was no difference between WT (N = 5) and *Stxbp1*$^{+/-}$ mice (N = 5) (3 hours recordings) (Unpaired *t*-test, t_8 = 0.4995, P = 0.6309). **d, e,** Distributions of length (**d**) and spike numbers (**e**) of SSC-SWDs during 3600-s recordings from five *Stxbp1*$^{+/-}$ mice. **f,** Local generation of SWDs examined by bipolar recordings. SSC-ECoG and SSC- and CPu-LFPs are monopolar recordings referenced to the cerebellum, whereas SSC- and CPu-bipolar recordings using bundled adjacent electrodes can detect local electrical signals by removing passive volume conduction of the electrical signals. All signals were recorded simultaneously during an SWD episode. **g,** Time-frequency plot (bottom) of ECoG recordings from an *Stxbp1*$^{+/-}$ mouse during SWDs (top) dominated by 6 Hz component (arrow). ECoG power spectrum density is pseudo-color coded.

Supplementary Fig. 2. Temporal/Phase relationships of SWDs in multiple brain regions in *Stxbp1*$^{+/-}$ mice.
a, Averaged spontaneous SWDs (left) and constant phase relationships among SSC-ECoG, mPFC-LFP, CPu-LFP and Thal-LFP recordings (enlarged in the dotted box; right) in *Stxbp1*$^{+/-}$ mice (N = 5) comprising 87 SWD events. **b,** Time delay of the first peak of mPFC, CPu and thalamic LFPs referenced to negative spike of SWDs in ECoG recordings. For each *Stxbp1*$^{+/-}$ heterozygote mouse (N = 5), SWDs of ECoG recordings and simultaneous LFP (mPFC, CPu and thalamus) recordings were aligned using a spike-triggered averaging method, and the time delay between the negative spike of ECoG SWDs (t = 0) and peaks of mPFC, CPU, and thalamic LFPs was estimated. There were

similar time delays in peaks for mPFC, CPu and Thal LFPs referenced to the negative spike of ECoG SWDs (Mann-Whitney *U* test; mPFC vs. CPu: P = 1.000; CPu vs. Thal: P = 0.6654; and mPFC vs. Thal: P = 1.000). **c,** Simultaneous spontaneous SWD recordings in an *Stxbp1*$^{+/-}$ mouse. **d,** Simultaneous spontaneous SSC- and CPu-SWDs recordings of absolute amplitude normalized against baseline activity (−5 to 0 s). The increase in CPu-SWD amplitude (red) was faster than that of SSC-SWD amplitude (black). Measurements in *Stxbp1*$^{+/-}$ mice (N = 5) comprising 87 SWD events. Inset: normalized activities of each SWD (−0.5 to −0.035 s; parentheses). The first spike of an SSC-SWD indicated by a dashed line (**c**) was used as a time reference (t = 0 s) in (**d**) **(**Mann–Whitney *U* test, ECoG vs. CPu, ***P < 0.0001).

Supplementary Fig. 3. Jumps in *Stxbp1*$^{+/-}$ and *Stxbp1*$^{fl/+}$/*Vgat* mice associated with spikes with positive deflection.
Sudden jumps in *Stxbp1*$^{+/-}$ (**a**) or *Stxbp1*$^{fl/+}$/*Vgat* (**b**) mice concurred with spikes with positive deflections in ECoG (SSC) recordings.

Supplementary Fig. 4. SWDs during awake, REM and non-REM sleep states in *Stxbp1*$^{+/-}$ mice. Representative SWDs during awake (W), non-REM (NREM), and REM in an *Stxbp1*$^{+/-}$ mouse. (Right) The occurrence of SWD number in each W or sleep state in *Stxbp1*$^{+/-}$ mice (N = 6, 24 hours recordings). SWDs probability (SWDs/hour); W: 13.6 ± 5.6, NREM: 5.9 ± 1.8, and REM: 41.9 ± 15.2. W time: 45.0 ± 2.5%, NREM sleep time: 45.9 ± 1.9%; and REM sleep time: 9.1 ± 0.6% in 24 hours. Data represent the mean ± SEM.

Supplementary Fig. 5. CPu activation causes generalized tonic-clonic seizures in *Stxbp1*$^{+/-}$ mice.
a, Schematic illustration of cannula placement for microinjection into the CPu (coronal section, Bregma 0.0, left hemisphere). Diffusion of red fluorescent dye (dextran tetramethylrhodamine; MW: 10000; 1.0 μl) within the striatum (blue: DAPI staining) 60 min after dye microinjection. **b,** Bicuculline injection into the CPu of *Stxbp1*$^{+/-}$ mice caused generalized tonic-clonic seizure. All tested *Stxbp1*$^{+/-}$ mice (N = 6) showed seizures shortly after bicuculline injections.

Supplementary Fig. 6. Temporal/Phase relationships of SWDs in *Stxbp1*$^{+/-}$ mice with electrical stimulation at the CPu.
Averaged electrical-induced SWDs (left) and their constant phase relationship (t = 0 s for electrical stimulation; right) at 60 ms after electrical stimulation (dotted box) from 431 events in an *Stxbp1*$^{+/-}$ mouse. Note the similarities between spontaneous (Supplementary Fig. 2a) and induced SWDs according to their phase relationships and polarity. Electrical microsimulations were 200 μs in duration.

Supplementary Fig. 7. Inactivation of the CPu or thalamus suppresses SWDs in *Scn2a*$^{fl/+}$/*Emx* mice.

Muscimol injection into the CPu or Thal (contralateral to the SSC-ECoG recording site) significantly reduced SWDs in *Scn2a*$^{fl/+}$/*Emx* mice. The number of SWDs for 9000 s after injection was quantified. [*Scn2a*$^{fl/+}$/*Emx* mice (N =3), unpaired *t*-test, vehicle vs. muscimol; CPu: t_4 = 5.41, **P = 0.0057; Thal: t_4 = 9.977, ***P = 0.0006]. The numbers of mice are indicated in parentheses.

Supplementary Fig. 8. *Trpc4*-dependent Cre expression in cortico-striatal projections and SWDs in *Scn2a*$^{fl/fl}$/*Trpc* mice.
a, Dominant expression of tdTomato (red) in cortical layer 5 neurons (coronal section) of a *Trpc4*-Cre mouse crossed with a tdTomato reporter mouse. **b,** tdTomato expression in cortico-striatal fibers in the CPu (coronal section). **c,** Spontaneous SWDs in a *Trpc4*-Cre dependent *Scn2a* conditional knockout mouse (*Scn2a*$^{fl/fl}$/*Trpc*).

Supplementary Fig. 9. Viral vectors used for the NeuRet system and their validation.
a, Synthetic sequence of the FLP-dependent fDIO cassette and Cre gene. **b,** NeuRet vector construction (top) and NeuRet vector with enhanced green fluorescent protein (middle). FLPe: a variant of the FLP recombinase; LTR: long terminal repeat; MSCVU3: U3 region of the LTR promoter of the murine stem cell virus; WPRE; woodchuck hepatitis virus posttranscriptional regulatory element; rBG: rabbit β globin poly(A) signals. AAV (serotype 5) vector construction (bottom). CAG pr: CAG promotor, ITR: inverted terminal repeat; Frt, F5: FLP recognition target sites; hGH: human growth hormone poly(A) signals. **c,** *In vitro* assay of FLPe-dependent Cre expression and Cre-dependent mCherry expression in Hek293FT cell culture. Co-transfection of plasmid CL20c-FLPe-IRES-EGFP, plasmid AAV-CAGGS-fDIO-Cre and plasmid AAV-EF1a-DIO-mCherry using lipofection. At 36 hours post-transfection, we observed fluorescence proteins indicating activity of the FLP and Cre recombinases. Induction of red fluorescence indicates that the FLP/Frt and Cre/loxP recombination processes worked properly (left). Images were taken at 36 hours post-lipofection.

Supplementary Fig. 10. **Electrophysiological and immunohistochemical identification of FSI and MSN.**
a, b, Electrophysiological characteristics of FSI (a) and MSN (b) *in vitro* and representative images of cells following injection of 1% biocytin (red), subsequent PV (green) immunostaining and DAPI (blue) staining. FSIs and MSNs exhibited fast spiking (>200 Hz) and slower spiking (<100 Hz) induced by depolarizing current injection, respectively. FSI identification was based on electrophysiological criteria [i.e. fast spiking (>200 Hz) with a brief spike width (<1ms), clear afterhyperpolarization, minimal firing adaptations, and low input resistance], although 6 cells were PV-positive and 6 cells were PV-negative among 12 cells displaying fast spiking. Scale bars: 25 μm.

Supplementary Fig. 11. Assessment of neocortical-striatal excitatory transmission in *Stxbp1*$^{+/-}$ and *Scn2a*$^{+/-}$mice.
a, Normalized EPSCs in striatal neurons induced by cortical stimulation in *Stxbp1*$^{+/-}$

littermate brain slices (10 MSNs and 9 FSIs, 19 slices from 9 WT mice; 10 MSNs and 9 FSIs, 19 slices from 9 *Stxbp1*$^{+/-}$ mice) (20 and 40 Hz). Data for 10 Hz are presented in Fig. 5c. **b,** The absolute amplitudes of the first EPSC responses induced by cortical stimulation of MSNs and FSIs in *Stxbp1*$^{+/-}$ mice [WT: 10 MSNs and 9 FSIs (N = 9); *Stxbp1*$^{+/-}$: 10 MSNs and 9 FSIs (N = 9); unpaired *t*-test, WT vs. *Stxbp1*$^{+/-}$, MSN: t_{18} = 0.5495, P = 0.5894; FSI: t_{16} = 0.7187, P = 0.4827]. **c,** The absolute amplitudes of asynchronous miniature EPSCs in MSNs and FSIs from *Stxbp1*$^{+/-}$ littermates under normal (buffer-containing Ca^{2+}) and release facilitated (Ca^{2+} replaced with Sr^{2+}) conditions. Box-and-whisker plot (whiskers: min to max). WT MSN Ca^{2+}, 211 events (5 mice, 9 cells); *Stxbp1*$^{+/-}$ MSN Ca^{2+}, 315 events (6 mice, 10 cells); WT MSN Sr^{2+}, 339 events (5 mice, 9 cells); *Stxbp1*$^{+/-}$ MSN Sr^{2+}, 442 events (6 mice, 10 cells); WT FSI Ca^{2+}, 874 events (7 mice, 8 cells); *Stxbp1*$^{+/-}$ FSI Ca^{2+}, 1094 events (5 mice, 9 cells); WT FSI Sr^{2+}, 987 events (7mice, 8 cells); and *Stxbp1*$^{+/-}$ FSI Sr^{2+}, 1131 events (5 mice, 9 cells). There were no statistical differences between WT and *Stxbp1*$^{+/-}$ cells (nested ANOVA, WT vs. *Stxbp1*$^{+/-}$, MSN, Ca^{2+}: P = 0.6387, Sr^{2+}: P = 0.4022; FSI, Ca^{2+}: P = 0.1881, Sr^{2+}: P = 0.4846. **d,** Normalized EPSCs in striatal neurons induced by cortical stimulation at 10 Hz in *Scn2a*$^{+/-}$ littermate brain slices (9 MSNs and 5 FSIs, 14 slices from 7 WT mice; 8 MSNs and 9 FSIs, 17 slices from 8 *Scn2a*$^{+/-}$ mice). **e,** EPSCs at steady-state (1,000 ms after stimulation) in *Scn2a*$^{+/-}$ littermate brain slices (WT: 9 MSNs and 5 FSIs; *Scn2a*$^{+/-}$: 8 MSNs and 9 FSIs). No statistically significant differences between WT and *Scn2a*$^{+/-}$ mice were observed (unpaired *t*-test, WT vs. *Scn2a*$^{+/-}$, MSN, 10 Hz: t_{15} = 1.527, P = 0.476; 20 Hz: t_{15} = 1.301, P = 0.2129; 40 Hz: t_{15} = 2.080, P = 0.0551; FSI, 10 Hz: t_{12} = 1.251, P = 0.2348; 20 Hz: t_{12} = 0.2297, P = 0.8222; 40 Hz: t_{12} = 0.3481, P = 0.7338. Mean ± SEM (**a,d,e**)

Supplementary Fig. 12. Estimation of RRP of synaptic vesicles in *Stxbp1*$^{+/-}$ mice.
a, Estimated RRP of synaptic vesicles was based on a cumulative curve of EPSC amplitude at 40 Hz stimulation. The Y-intercept (arrow) of the linear extrapolation from data points at the 30th to the 40th stimulations corresponds to the size of the RRP in cortico-striatal synapses. **b,** Estimated RRPs were similar between WT and *Stxbp1*$^{+/-}$ mice (10 MSNs and 9 FSIs, 19 slices from 9 WT mice; 10 MSNs and 9 FSIs 19 slices from 9 *Stxbp1*$^{+/-}$ mice) (unpaired *t*-test, MSN: t_{18} = 0.1873, P = 0.8535; FSI: t_{17} = 0.7892, P = 0.4409). RRPs for FSIs were larger than those of MSNs in both genotypes (Mann–Whitney *U* test, WT: ***P = 0.0002; *Stxbp1*$^{+/-}$: *P = 0.0191). **c,** Estimated release probabilities were similar between WT and *Stxbp1*$^{+/-}$ mice (unpaired *t*-test, MSN: t_{18} = 0.3069, P = 0.7624; FSI: t_{17} = 1.501, P = 0.1518). *P < 0.05, ***P < 0.001.

Supplementary Fig. 13. NASPM injections into the CPu or nRT induce epileptic activities in mice.
a, NASPM (0.5 μl; bilateral) injected into the CPu of WT mice induced continuous SWD-like activity in SSC-ECoG recordings before the appearance of convulsive seizures (2 of 6 mice). **b,** NASPM (1.0 μl; bilateral) injected into the nRT similarly induced generalized convulsive seizures associated with epileptic ECoG and LFPs (N = 2).

Supplementary Fig. 14. Gq DREADD vector and suppression of SWDs in *Stxbp1*$^{+/-}$ mice by CNO intraperitoneal injection.
a, AAV construction for the DREADD system. The vector contains a double-floxed inverse open reading frame (DIO) and hM3D (Gq) for activating neuronal activity. **b,** Immunolabeled PV-positive FSIs (green; arrow heads) in the dorsal striatum of an *Stxbp1*$^{+/-}$ mouse >4 weeks after AAV injection (coronal section; left). Cre-dependent mCherry (red; arrow heads) expression indicates the expression of DREADD receptors (middle). Merged image of PV and mCherry expressions (right). Scale bar: 100 µm. **c,** SWDs were suppressed after intraperitoneal administration of CNO (1 mg/kg) (3 hours recordings post-injection). *Stxbp1*$^{+/-}$/PV Gq with AAV injection in the CPu (N = 5), vehicle vs. CNO, unpaired *t*-test, $t_8 = 4.491$, **P = 0.0020. **P < 0.01.

Supplementary Fig. 15. **Striatal MSN and FSI neuronal activities at the onset of SWDs in *Stxbp1*$^{+/-}$ mice.**
a, Spike clustering and classification based on spike waveforms. Each point corresponds to a neural spike represented in a feature space (e.g., principal components, peak-valley amplitude, etc.). One cluster (yellow) corresponds to a single unit of pFSI, and the other cluster (green) corresponds to pMSN simultaneously recorded from a single electrode. PCA: principal component analysis. **b,** Parameters used for spike classification, including spike half-width and peak-valley duration. **c,** Scatter plot of spike half-width versus peak-valley duration in the single units. The dotted horizontal line divides the units with narrow spike widths and the units with broad spike widths [WT (N = 3), 160 cells; *Stxbp1*$^{+/-}$ (N = 10), 277 cells]. **d,** There was no statistical difference between WT and *Stxbp1*$^{+/-}$ mice in firing rates of pMSNs and pFSIs during the recording sessions. MSN: WT (89 cells) vs. *Stxbp1*$^{+/-}$ (93 cells), unpaired *t*-test, $t_{185} = 1.239$, P = 0.2171; FSI (>6 Hz): WT (6 cells) vs. *Stxbp1*$^{+/-}$ (18 cells), $t_{22} = 1.986$, P = 0.0596. **e, f,** Scatter plot of the firing rates of single pFSI (**e**) and pMSN (**f**) units at baseline (−5 to −4 s before SWD onset) and onset (−0.5 to +0.5 s at SWD onset). **g,** Firing rates (baseline and SWD onset) of pFSIs that have firing rates >6, >8, and >10 Hz or non-FSIs <6 Hz at baseline. We consistently observed that pFSIs firing rate declined at the onset of SWDs as compared with baseline firing [baseline vs. onset, Mann-Whitney *U* test: pFSIs >10 Hz (N = 9), mean firing rate 12.88 Hz, P = 0.0503; pFSIs >8 Hz, N = 13, mean firing rate 11.62 Hz, P = 0.0727; pFSIs >6 Hz, N = 18, mean firing rate 10.47 Hz, *P = 0.0279; non-FSIs <6 Hz, N = 15, mean firing rate 2.288 Hz, P = 0.6187]. **h,** An example of pFSI spiking activity during an SWD episode. Such oscillatory activities were observed in 3 out of 18 pFSIs. Numbers of cells are indicated in parenthesis (**d, e, f, h**). Mean ± SEM.

Supplementary Video 1. Spontaneous tonic-clonic seizure in an *Stxbp1*$^{+/-}$ mouse.
Video length: 35 s.

Supplementary Video 2. Epileptic jump of an *Stxbp1*$^{+/-}$ mouse.
Video length: 6 s.

Supplementary Video 3. Epileptic jump of an *Stxbp1*$^{fl/+}$/*Vgat* mouse.
Video length: 4 s.

Supplementary Video 4. Generalized tonic-clonic seizure in a mouse injected with NASPM in the CPu.
Video length: 24 s [36 min after bilateral injection of NASPM (5 mM; 1.0 µl) into a WT mouse].

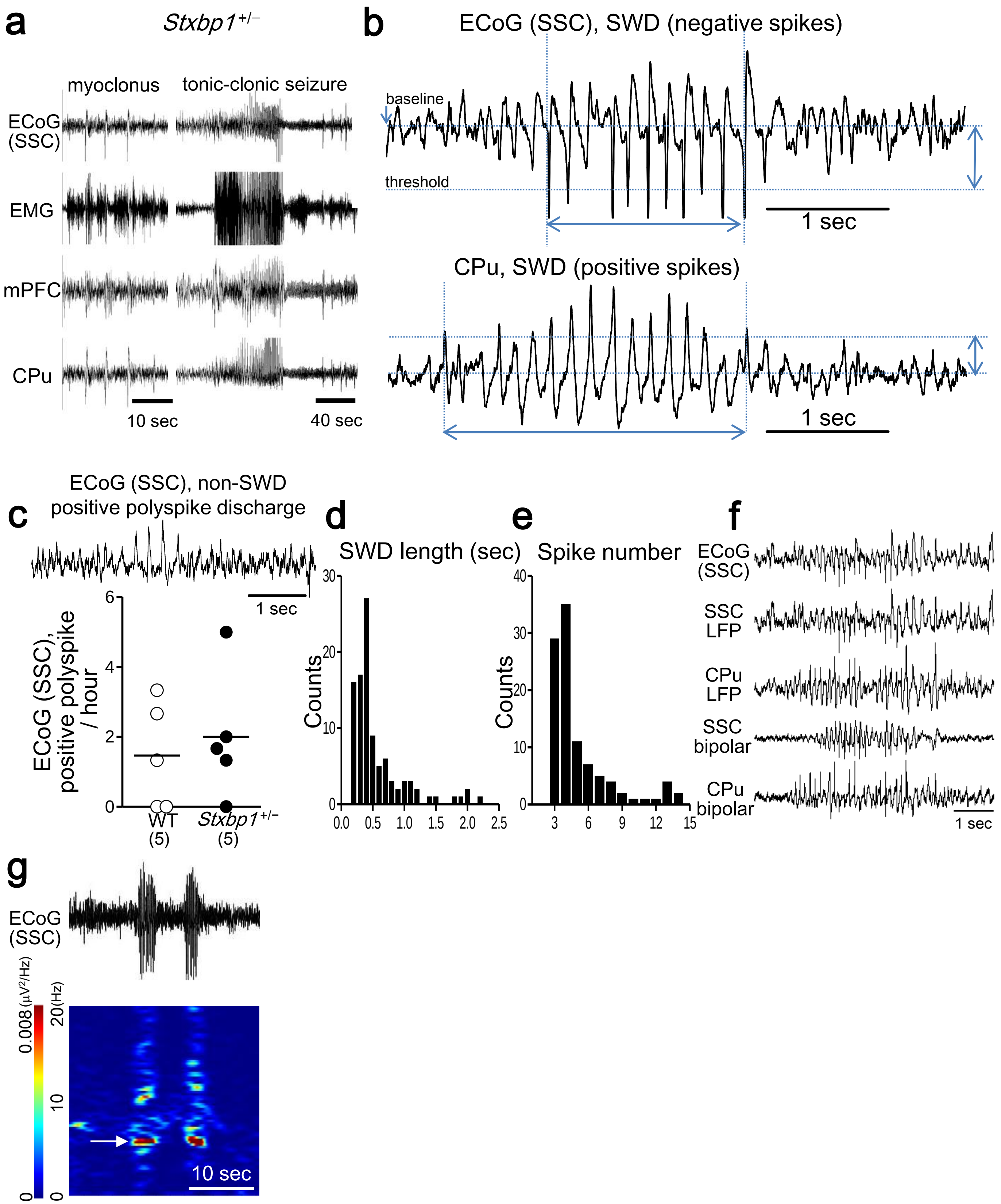

Miyamoto Supplementary Figure 1

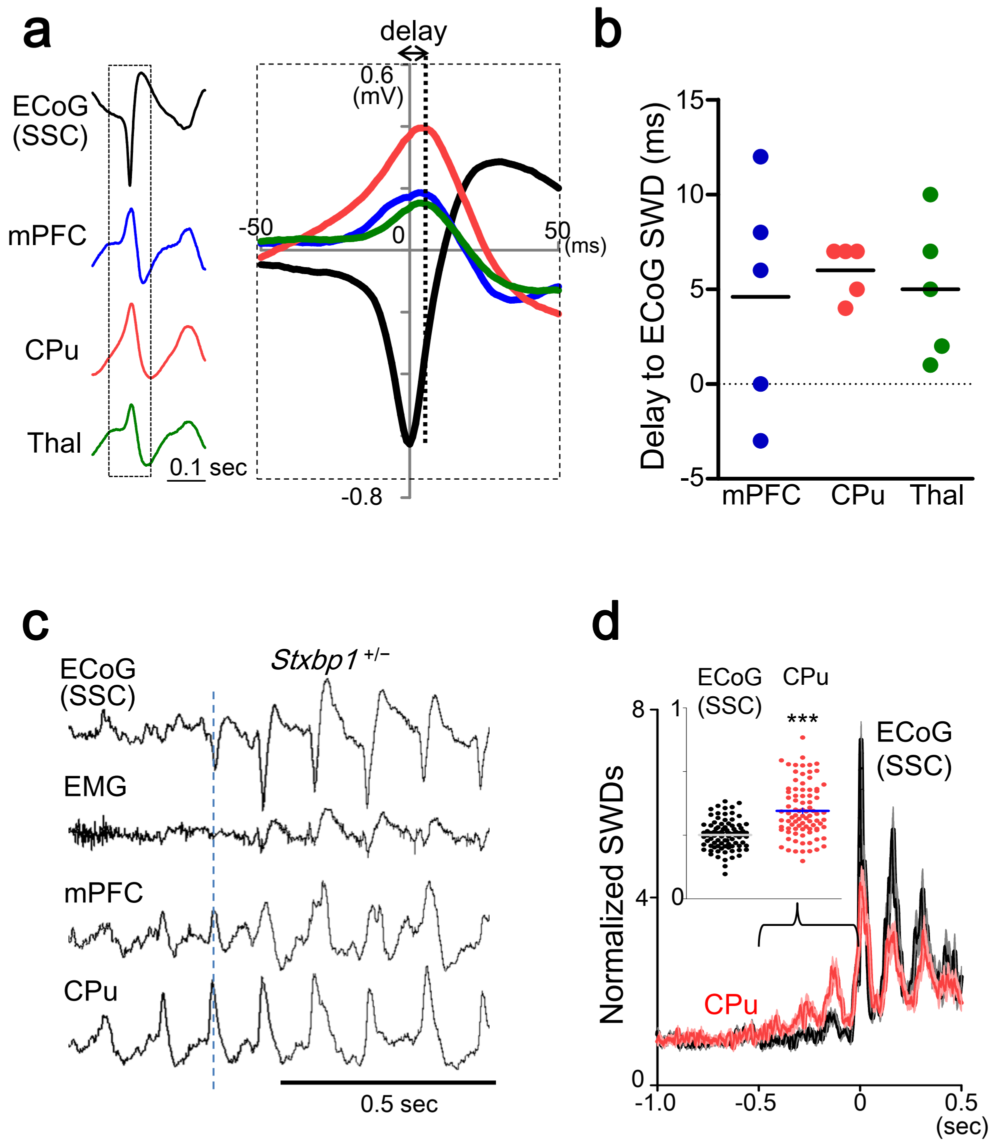

Miyamoto Supplementary Figure 2

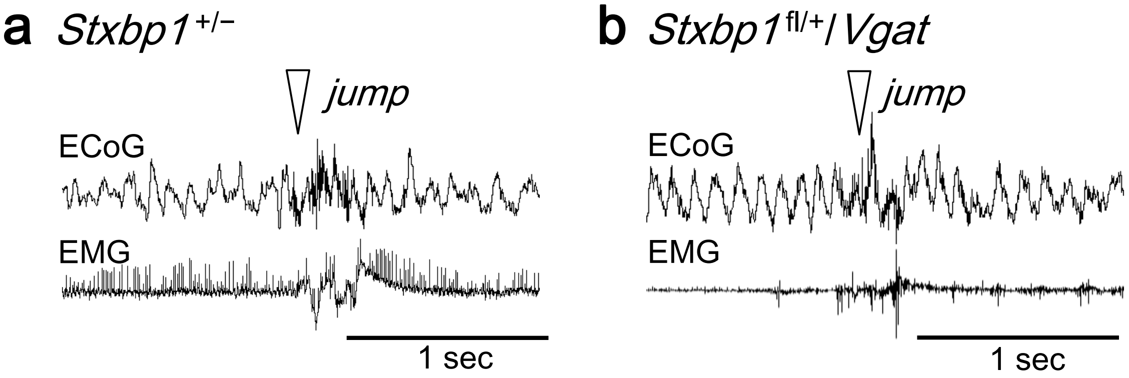

Miyamoto Supplementary Figure 3

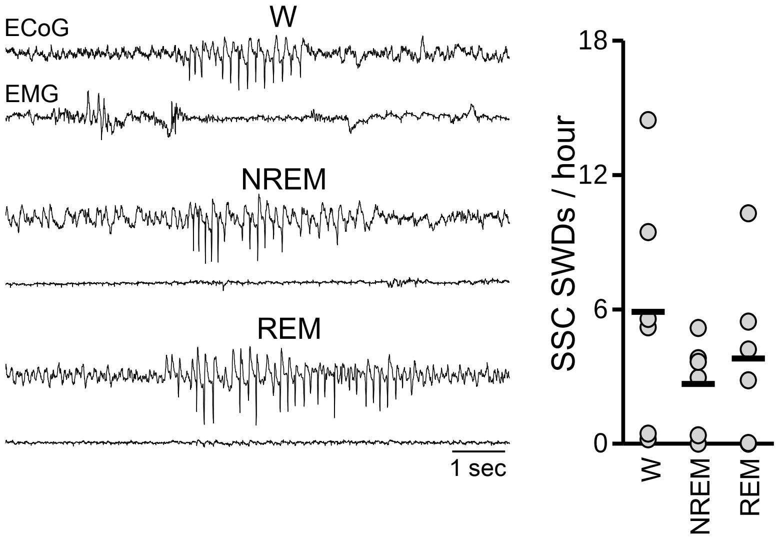

Miyamoto Supplementary Figure 4

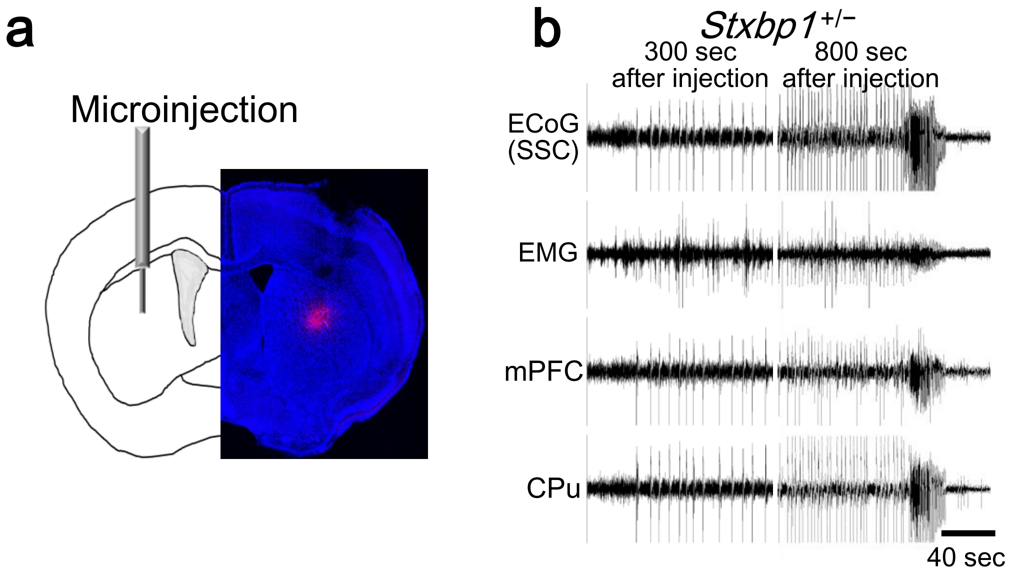

Miyamoto Supplementary Figure 5

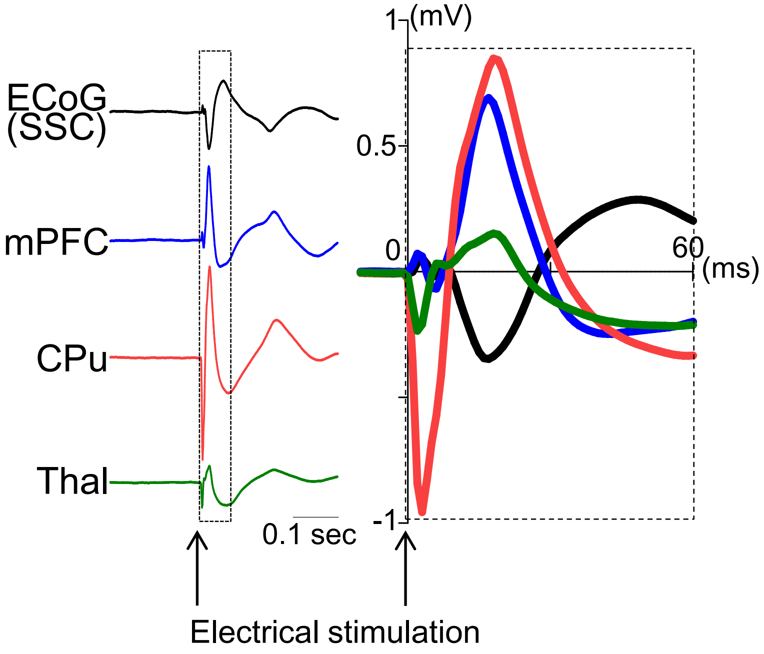

Miyamoto Supplementary Figure 6

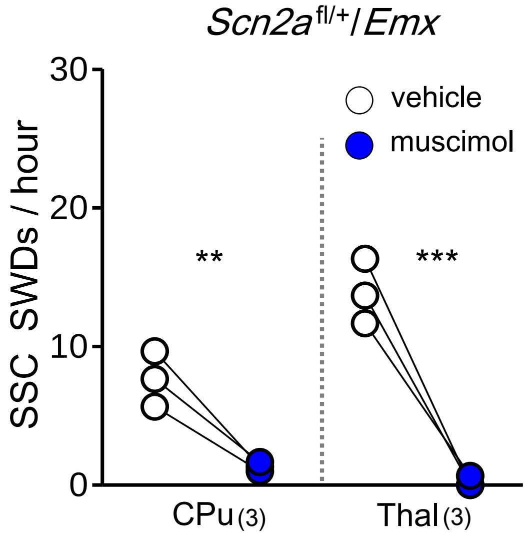

Miyamoto Supplementary Figure 7

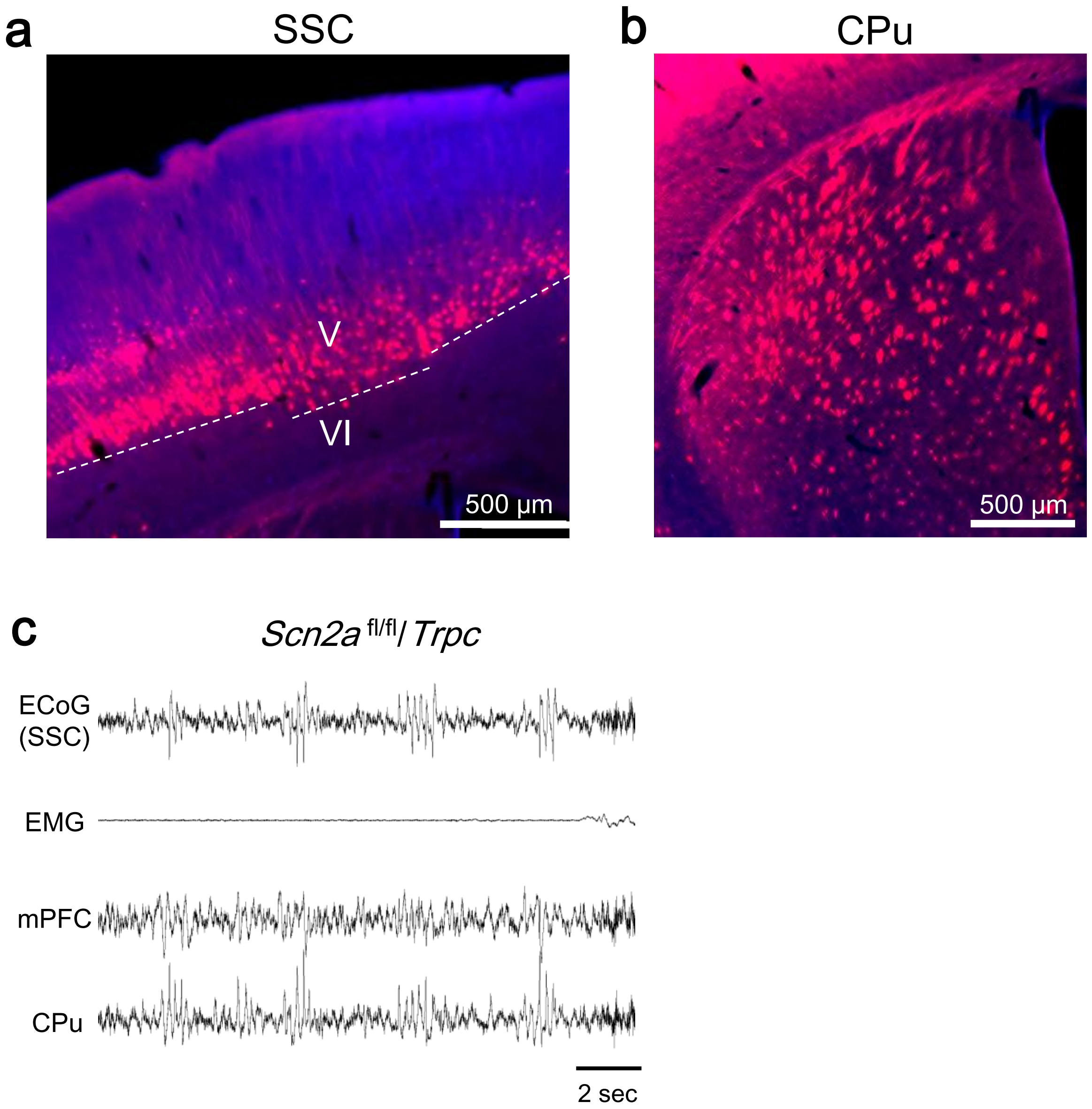

Miyamoto Supplementary Figure 8

a Synthetic sequence of fDIO cassette and Cre gene

Frt / F5

```
ggatcctctagagtcgactccggagaagttcctattctctagaaagtataggaacttcgcagaatggtagctggattgtagctgct
attagcaatatgaaacctcttagaagttcctattcttcaaaaggtataggaacttcggcgcgcc
```

Cre

```
TCAGTCCCCA TCCTCGAGCA GCCTCACCAT GGCCCCAGTC TCAGAGTCCA GGTTTCTGAT GTAGTTCATC ACAATGTTCA
CATTGGTCCA GCCACCAGCC TGCATGATTT CAGGGATGGA CACACCAGCC CTGGCCATGT CCCTGGCAGC ACCCACTCTG
GCAGAGTGGC CAGACCAGGC CAGGTATCTC TGCCCAGAGT CATCCTTGGC ACCATAGATC AGGCGGTGGG TGGCCTCAAA
GATCCCTTCC AGGGCCCGGG TGGACAGTTG GGAGGTGGCA GAAGGGGCAG CCACACCATT CTTTCTGACC CGGCAGAACA
GGTAGTTGTT GGGGTCATCA GCCACACCAG ACACAGAGAT CCATCTCTCC ACCAGCTTGG TAACCCCCAG GGACAGGGCC
TTCTCCACAC CAGCTGTGGA CACCAGGGTC TTGGTCCTGC CAATGTGGAT CAGCATTCTC CCACCATCGG TGCGGGAGAT
GTCCTTCACT CTGATTCTGG CAATTTCGGC AATGCGCAGC AGGGTGTTGT AGGCAATGCC CAGGAAGGCC AGGTTCCTGA
TGTCCTGGCA TCTGTCAGAG TTCTCCATCA GGGATCTGAC TTGGTCAAAG TCAGTGCGTT CAAAGGCCAG GGCCTGCTTG
GCTCTCTCCC CAGCATCCAC ATTCTCCTTT CTGATTCTCC TCATCACCAG GGACACAGCA TTGGAGTCAG AAGGGCGAGG
CAGGCCAGAT CTCCTGTGCA GCATGTTGAG CTGGCCCAGG TGCTGTTGGA TGGTCTTCAC AGCCAGGCCT CTGGCTTGCA
GGTACAGGAG GTAGTCCCTC ACATCCTCAG GTTCAGCAGG GAACCATTTC CTGTTGTTCA GCTTGCACCA GGCAGCCCAG
GATCTGCACA CAGACAGGAG CATCTTCCAG GTGTGTTCAG AGAAGGCCTG CCTGTCCCTG AACATGTCCA TCAGGTTCTT
CCTGACTTCA TCAGAGGTGG CATCCACAGG GAGGGCAGGC AGGTTTTGGT GCACAGTCAG CAGGTTGGAG ACTTTCCTCT
TCTTCTTGGG CACCAT
```

Frt / F5

```
ggtggcgctagcgaagttcctatactttctagagaataggaacttcttgccttaacccagaaattatcactgttattctttagaatg
gtgcaaagagaagttcctataccttttgaagaataggaacttcgaattcgatatc
```

1348 bp

b

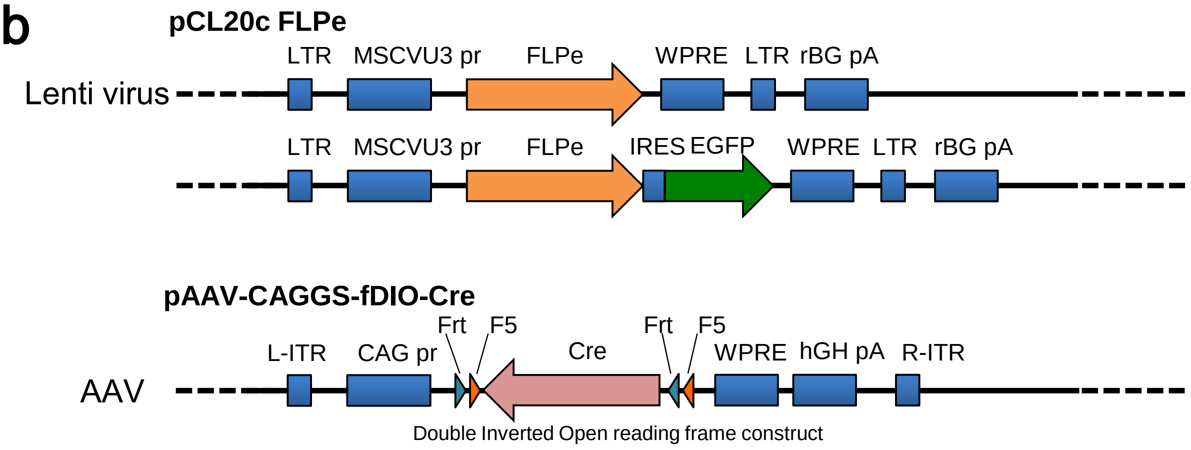

c

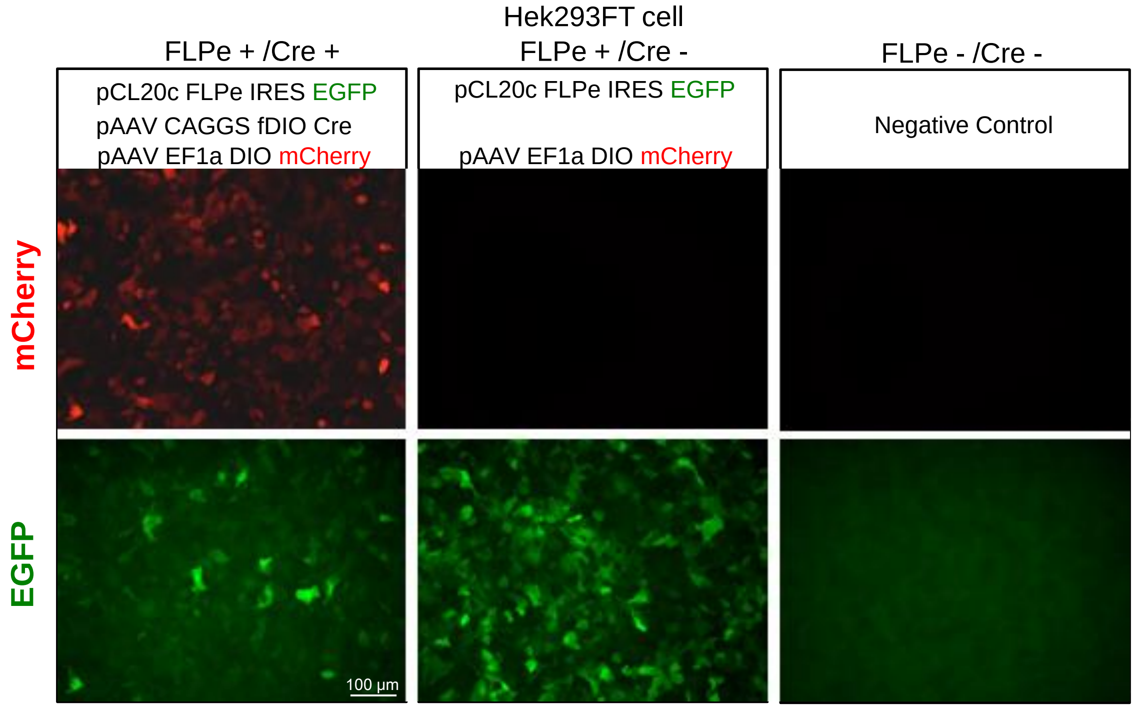

Miyamoto Supplementary Figure 9

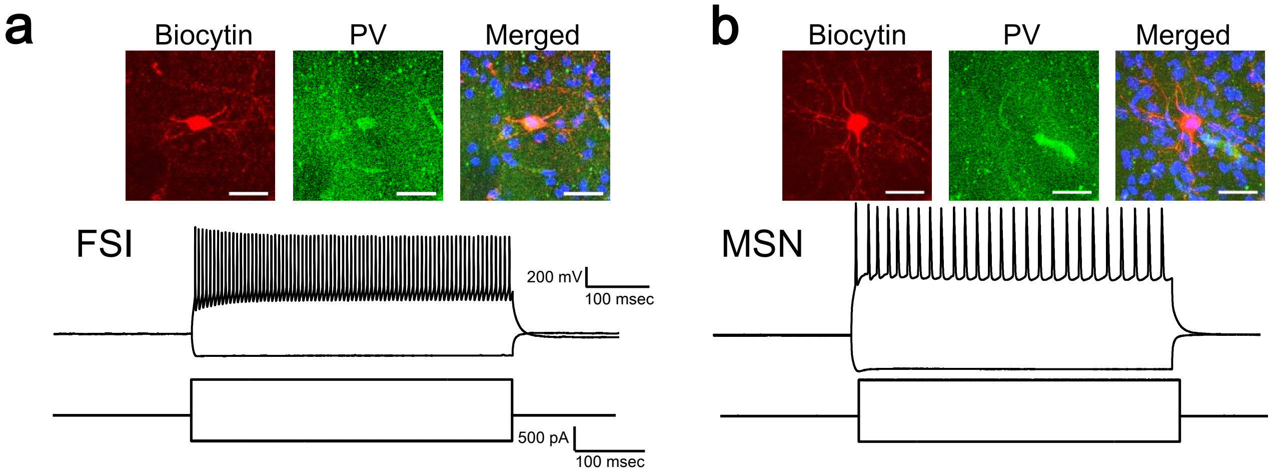

Miyamoto Supplementary Figure 10

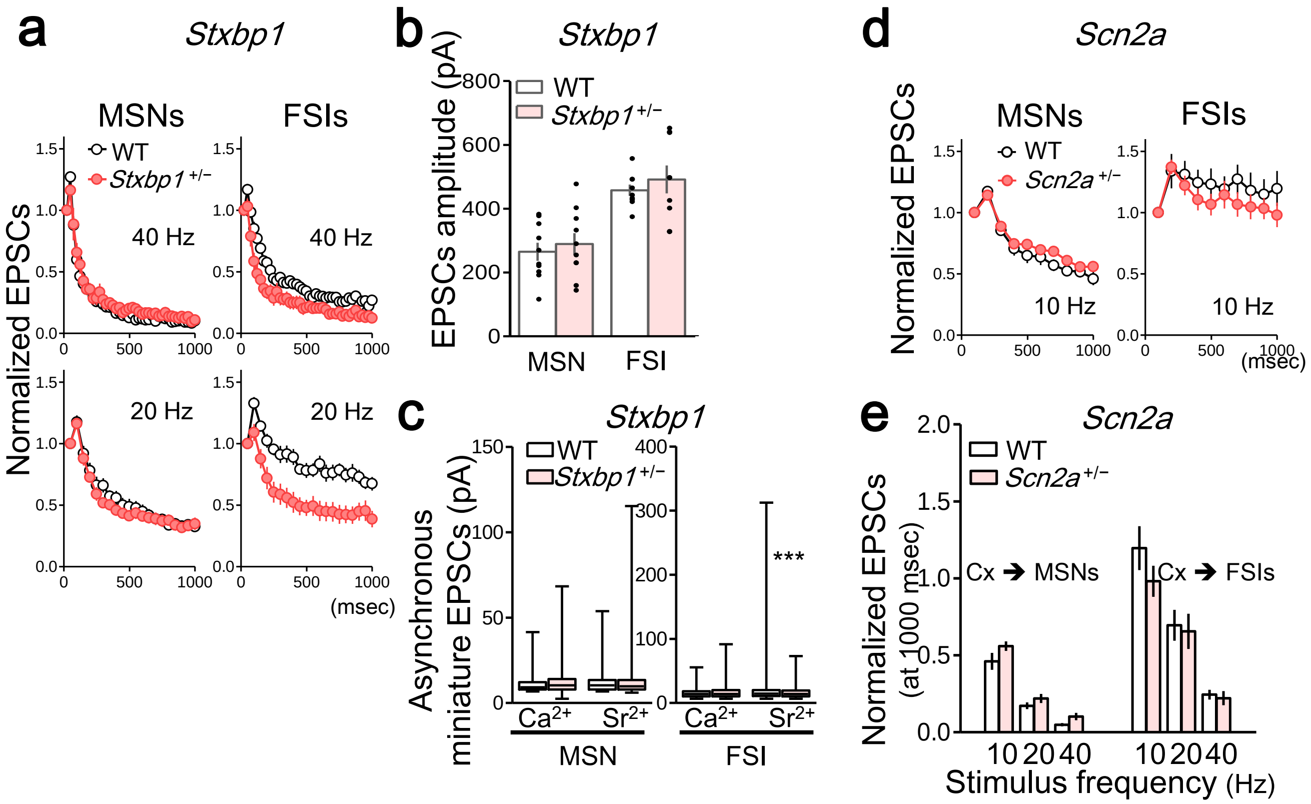

Miyamoto Supplementary Figure 11

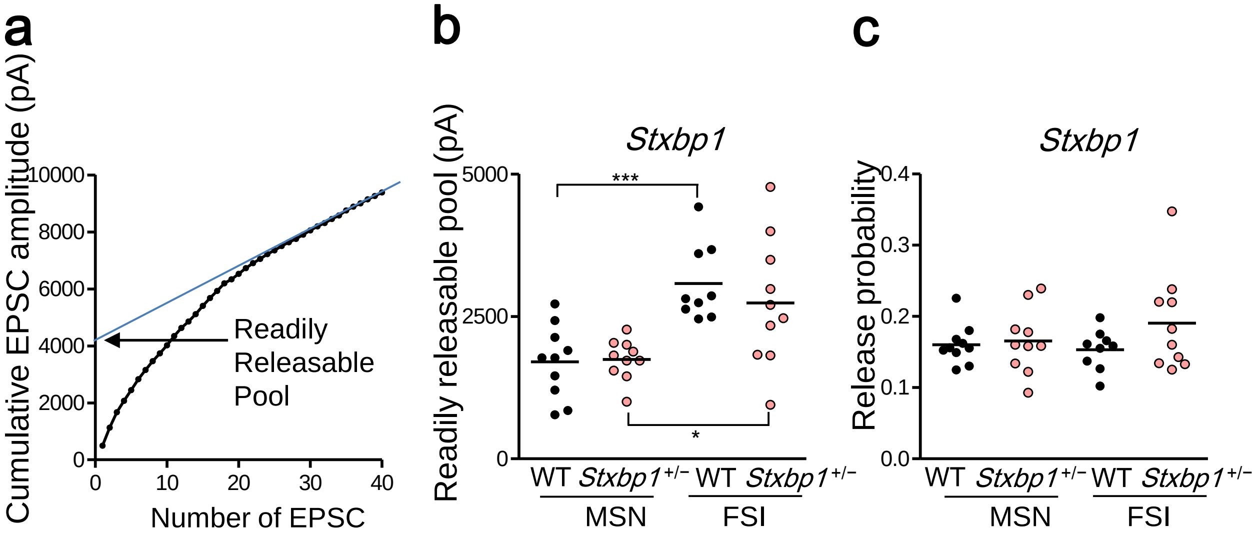

Miyamoto Supplementary Figure 12

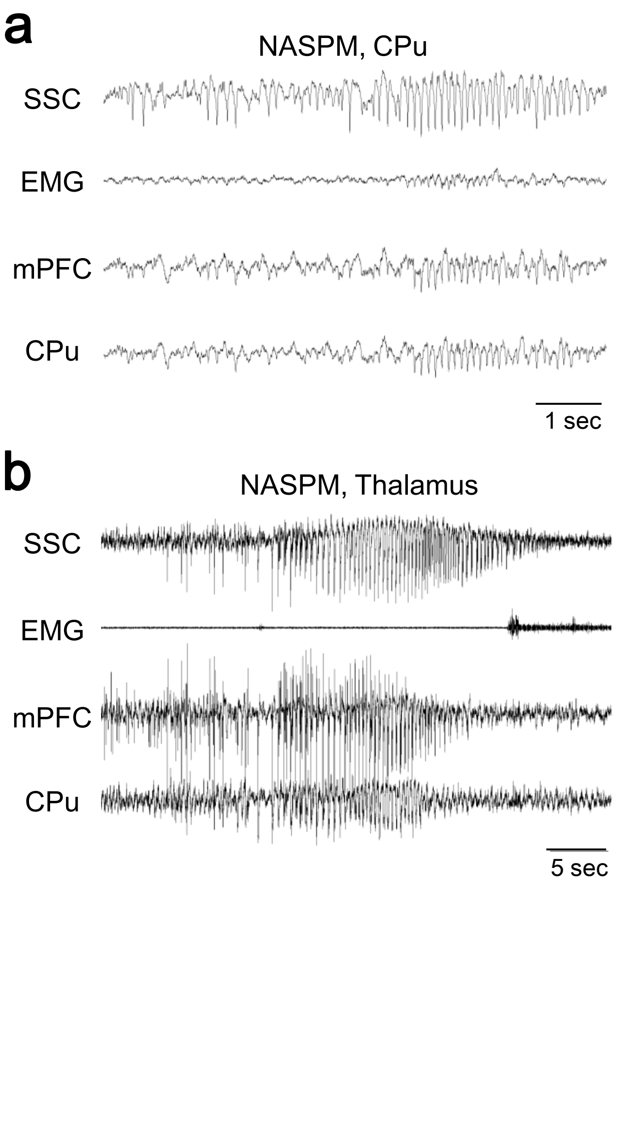

Miyamoto Supplementary Figure 13

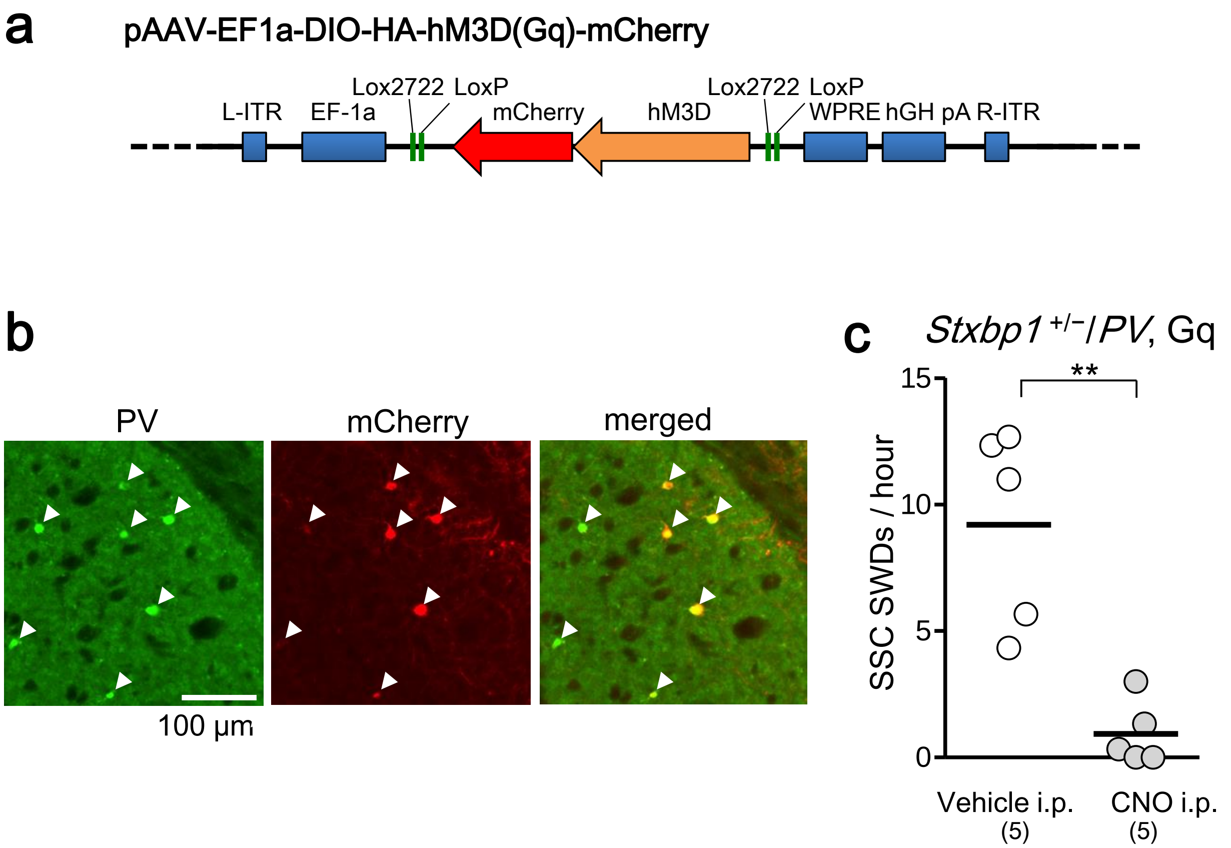

Miyamoto Supplementary Figure 14

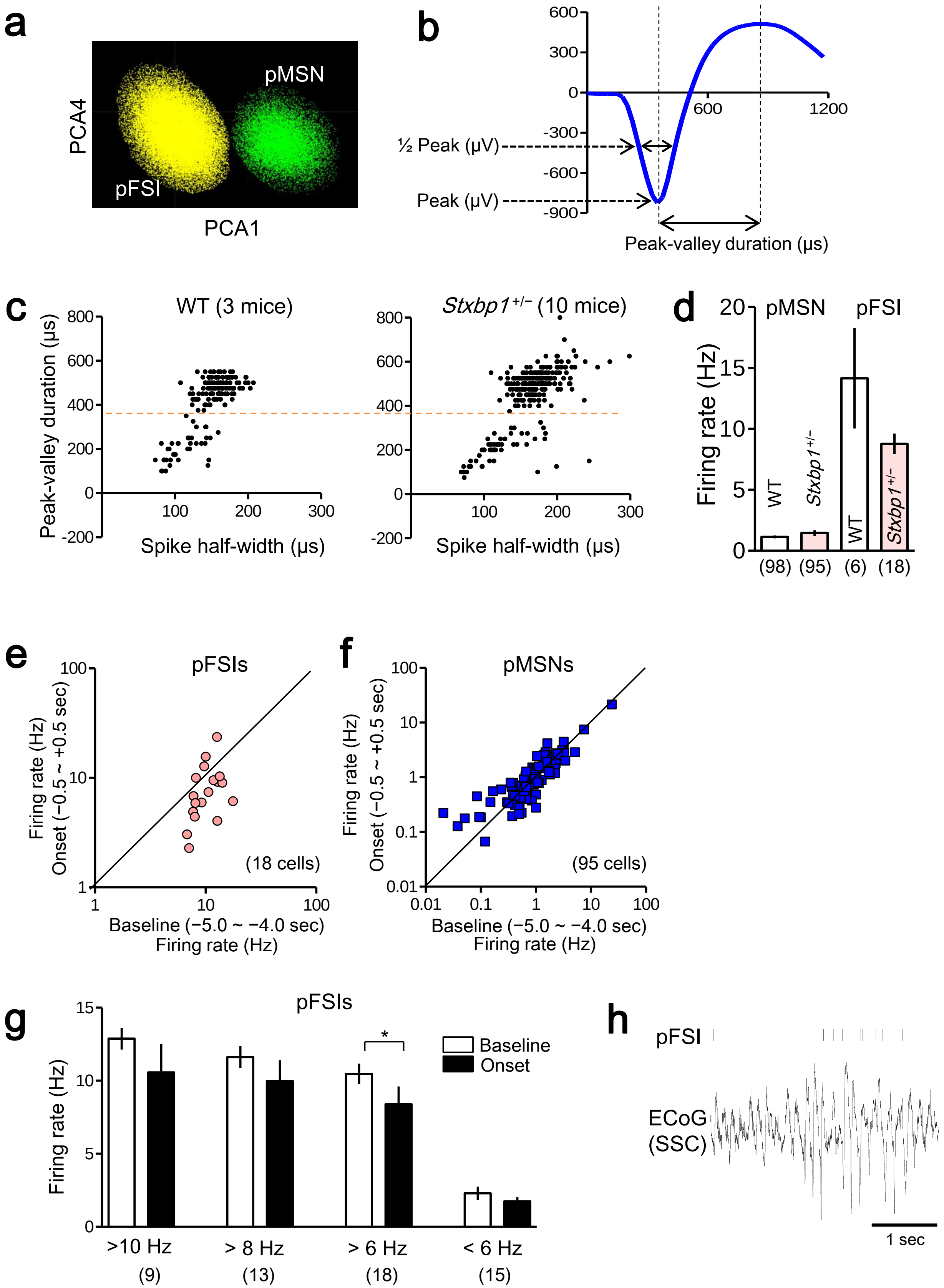

Miyamoto Supplementary Figure 15
