# Supplementary material for: Impaired cortico-striatal excitatory transmission triggers epilepsy
Source: Nat Commun. 2019 Apr 23;10:1917. doi: 10.1038/s41467-019-09954-9 (PMC6478892; doi:10.1038/s41467-019-09954-9)
Supplement: Supplementary file 3 — Reporting Summary [file 41467_2019_9954_MOESM3_ESM.pdf]

nature research

Corresponding author(s): Kazuhiro Yamakawa

Reporting Summary

Nature Research wishes to improve the reproducibility of the work that we publish. This form provides structure for consistency and transparency in reporting. For further information on Nature Research policies, see Authors & Referees and the Editorial Policy Checklist.

Statistical parameters

When statistical analyses are reported, confirm that the following items are present in the relevant location (e.g. figure legend, table legend, main text, or Methods section).

n/a	Confirmed	
☐	☒	The exact sample size (*n*) for each experimental group/condition, given as a discrete number and unit of measurement
☐	☒	An indication of whether measurements were taken from distinct samples or whether the same sample was measured repeatedly
☐	☒	The statistical test(s) used AND whether they are one- or two-sided<br>*Only common tests should be described solely by name; describe more complex techniques in the Methods section.*
☐	☒	A description of all covariates tested
☐	☒	A description of any assumptions or corrections, such as tests of normality and adjustment for multiple comparisons
☐	☒	A full description of the statistics including central tendency (e.g. means) or other basic estimates (e.g. regression coefficient) AND variation (e.g. standard deviation) or associated estimates of uncertainty (e.g. confidence intervals)
☐	☒	For null hypothesis testing, the test statistic (e.g. *F*, *t*, *r*) with confidence intervals, effect sizes, degrees of freedom and *P* value noted<br>*Give P values as exact values whenever suitable.*
☒	☐	For Bayesian analysis, information on the choice of priors and Markov chain Monte Carlo settings
☒	☐	For hierarchical and complex designs, identification of the appropriate level for tests and full reporting of outcomes
☒	☐	Estimates of effect sizes (e.g. Cohen's *d*, Pearson's *r*), indicating how they were calculated
☐	☒	Clearly defined error bars<br>*State explicitly what error bars represent (e.g. SD, SE, CI)*

Our web collection on statistics for biologists may be useful.

Software and code

Policy information about availability of computer code

Data collection	VitalRecorder (Kissei) for ECoG/EMG data, MAP data acquisition system (Plexon) for ECoG/LFP/spike data, pCLAMP 10 (Molecular Devices) for in vitro electrophysiological data.
Data analysis	SleepSign (Kissei) for ECoG/EMG data analysis, NeuroExplorer 4 (Nex Technologies) for ECoG/LFP data data analysis, Offline Sorter 2.8.8 (Plexon) for spike sorting, Origin 8.5 J (Lightstone) for in vitro electrophysiological data analysis, GraphPad Prism 5 for statistical analysis.

For manuscripts utilizing custom algorithms or software that are central to the research but not yet described in published literature, software must be made available to editors/reviewers upon request. We strongly encourage code deposition in a community repository (e.g. GitHub). See the Nature Research guidelines for submitting code & software for further information.

Data

Policy information about availability of data

All manuscripts must include a data availability statement. This statement should provide the following information, where applicable:

- Accession codes, unique identifiers, or web links for publicly available datasets
- A list of figures that have associated raw data
- A description of any restrictions on data availability

All data supporting the findings of this study are available within the article and its supplementary information file or are available from the corresponding author upon reasonable request.

Field-specific reporting

Please select the best fit for your research. If you are not sure, read the appropriate sections before making your selection.

☒ Life sciences ☐ Behavioural & social sciences ☐ Ecological, evolutionary & environmental sciences

For a reference copy of the document with all sections, see nature.com/authors/policies/ReportingSummary-flat.pdf

Life sciences study design

All studies must disclose on these points even when the disclosure is negative.

Sample size	Sample size calculation was not performed. Sample size in each experiments was determined based on pilot studies (magnitude/direction of changes, variability, reproducibility etc.) and published experiments. Sample sizes are reported in figure legends.
Data exclusions	No animals in vivo experiments were excluded. In slice electrophysiology, data were discarded when series resistance exceeded > 20 MΩ or varied by more than ± 10%.
Replication	All key experiments were repeated with different litters to obtain sufficient number of mice.
Randomization	Mice were randomly assigned to treatments/experiments.
Blinding	Quantification of spike-and wave discharges were performed by the experimenters blind to mouse genotypes or experimental treatments.

Reporting for specific materials, systems and methods

Materials & experimental systems

n/a	Involved in the study	
☒	☐	Unique biological materials
☐	☒	Antibodies
☐	☒	Eukaryotic cell lines
☒	☐	Palaeontology
☐	☒	Animals and other organisms
☒	☐	Human research participants

Methods

n/a	Involved in the study	
☒	☐	ChIP-seq
☒	☐	Flow cytometry
☒	☐	MRI-based neuroimaging

Antibodies

Antibodies used	rabbit anti-parvalbumin antibody (catalog no. PC255L; Calbiochem, Merck Millipore)<br>donkey anti-rabbit IgG, Alexa Fluor 488-conjugated (catalog no. A21206, Thermo Fisher Scientific)
Validation	Information about antibody is described in the method section. The antibodies are validated by vendors and by our own experiments.

Eukaryotic cell lines

Policy information about cell lines

Cell line source(s)	HEK293FT cells (Thermo Fischer Scientific, R70007)
Authentication	Cell lines were authenticated by Thermo Fisher Scientific.
Mycoplasma contamination	The cell lines were not tested for mycoplasma contamination.
Commonly misidentified lines (See ICLAC register)	Commonly misidentified cell lines were not used.

Animals and other organisms

Policy information about studies involving animals; ARRIVE guidelines recommended for reporting animal research

Laboratory animals	Mus musculus: C57BL/6N or C57BL/6J, male and female, postnatal day 21-34 or over 2 months of age. Rattus norvegicus: GAERS/Mave, male and female over 4 months of age
Wild animals	The study did not involve wild animals that were captures in the field.
Field-collected samples	The study did not involve samples collected from the field.
