# Supplementary material for: Impaired cortico-striatal excitatory transmission triggers epilepsy
Source: Nat Commun. 2019 Apr 23;10:1917. doi: 10.1038/s41467-019-09954-9 (PMC6478892; doi:10.1038/s41467-019-09954-9)
Supplement: Supplementary file 4 — Description of Additional Supplementary Files [file 41467_2019_9954_MOESM4_ESM.pdf]

**Description of Additional Supplementary Files**

File Name: Supplementary Movie 1
Description: Spontaneous tonic-clonic seizure in an Stxbp1$^{+/-}$ mouse. Video length: 35 s.

File Name: Supplementary Movie 2
Description: Epileptic jump of an Stxbp1$^{+/-}$ mouse. Video length: 6 s.

File Name: Supplementary Movie 3
Description: Epileptic jump of an Stxbp1$^{fl/+}$ /Vgat mouse. Video length: 4 s.

File Name: Supplementary Movie 4
Description: Generalized tonic-clonic seizure in a mouse injected with NASPM in the CPu. Video length: 24 s [36 min after bilateral injection of NASPM (5 mM; 1.0 μl) into a WT mouse].
